# Incentives Promoting Contracted Family Doctor Service Policy to Improve Continuity and Coordination in Diabetes Patient Management Care in China

**DOI:** 10.3389/fpubh.2022.843217

**Published:** 2022-07-15

**Authors:** Yinzi Jin, Wenya Tian, Yahang Yu, Wen Pan, Beibei Yuan

**Affiliations:** ^1^Department of Global Health, School of Public Health, Peking University, Beijing, China; ^2^Institute for Global Health and Development, Peking University, Beijing, China; ^3^Department of Chronic Disease Epidemiology, School of Public Health, Yale University, New Haven, CT, United States; ^4^China Center for Health Development Studies, Peking University, Beijing, China

**Keywords:** contracted family doctor service, incentive, diabetes patient management care, continuity, coordination

## Abstract

**Background:**

As the first step toward building a gatekeeping system in China, the governments have introduced a contracted family doctor service (CFDS) policy in primary healthcare (PHC) facilities. This study was to examine the association between apply of incentive to improve the implementation of CFDS and the performance on diabetes management care.

**Methods:**

We conducted a cross-sectional study in 72 PHC facilities in 6 cities that piloted the CFDS. Multivariate regression models were applied, based on a sample of 827 PHC providers and 420 diabetic patients.

**Results:**

PHC providers who reported the performance being linked with increased income were 168.1 and 78.0% more likely to have good continuity and coordination of diabetes patient management care, respectively. Additional one-point percentage of PHC providers whose performance on CFDS was assessed was associated with 7.192 times higher probability of patients with control of blood glucose.

**Discussion:**

Inclusion of incentives rewarding better performance on CFDS were associated with better delivery process and outcome performance on diabetes management care.

**Conclusion:**

Design and implementation of the incentive should be accompanied with the policy of CFDS, in order to increase the proportion of performance-related income of PHC providers, thereby improving the quality of diabetes management care.

## Introduction

There were an estimated 422 million people suffering from diabetes, and up to 8.5% of the world's population aged over 18 years in 2014 (1). In China, the number of adult diabetes patients is as high as 114 million, ranking first globally, accounting for more than a quarter of the total number of the world's diabetes (2). The prevalence of diabetes in China is increasing from 0.9% in 1990 to 11.2% in 2017 (3). However, a nationally representative survey in 2013 found that only about 30% of patients with type 2 diabetes were being treated, among which 40% achieved glycemic control (4). The epidemic of diabetes is associated with high economic burden, impaired physical health, and reduced life quality, imposing a high burden on the adults (5).

The successful management of type 2 diabetes requires continuity and coordination of care. Continuity of care needs patient's “continuous caring relationship” with an identified healthcare provider. Coordination of care refers to the delivery of a “seamless services” through the sharing of information between different departments inside a health facility or among different level of healthcare providers (6). Doctors and other health workers in primary healthcare (PHC) facilities are in a central position to coordinate the diabetes patients' care needs, from early detection to disease management (7). And the contractual arrangements between patients and primary healthcare doctors can be a facilitator for building stable relationship and continuous care. As the first step toward building a gatekeeping system in China, the governments have introduced a “contracted family doctor service” (CFDS) policy by which each resident would be registered with a team of PHC health workers including doctors, nurses and public health personals since 2016 (8). National government of China set goals for this policy, including achieving a 30% rate of residents covered by contract services by 2017 and universal coverage to be achieved by 2020 (8, 9). However, this policy is still in an early stage, and the scaling up has been hindered by several factors, including the low motivation of health workers in PHC facilities because of the weakness in design of financial incentives for contract services (10, 11).

In China, doctors and other health workers in PHC facilities are employees of PHC facilities, and they are paid by a performance-based salary system (PBS). In this payment system recommended by national policy guidance, total amount of salary included two parts: one basic salary (60–70% of the total income) and one performance-based bonus salary (30–40% of the total income) (7, 12). To further improve the incentives, later policies repeatedly mentioned “enlarge the variation of total income distribution among PHC facilities employees” and “appropriately increase the proportion of performance-based bonus salary in total salary.” Within allowed range of national policy documents, the specific design of PBS varied across areas and facilities in the same area, because current policies grant PHC facility the autonomy of setting proportion of performance-based part in total salary. It was reported that in practice, most of facilities used the quantity of services as the major criteria to calculate performance-based income rather than the quality of care delivered (7, 12).

A growing strand of literature has studied the aligning pay-for-performance incentive as an intervention for improving quality of diabetes patient management care (13–19). Pay-for-performance is a payment policy that provides financial incentives to healthcare providers based on specific predetermined quality benchmarks (20–22), and PBS for health workers in PHC facilities in China is also a kind of performance related payment targeting individual health workers. In 2009, China initiated basic public health services package and included diabetes patient management care in package, and in 2016 the “contracted family doctor service” policy was initiated and this policy prioritizes building the contracts with chronic disease patients. With the requirements of two major policies, more and more public health institutions introduced the performance on chronic disease management into performance assessment criteria and linking the assessment results to income of PHC providers with the purpose to motivate providers' behavior in the chronic disease management. However, less is known that whether change of performance assessment practice have started to work on change the behavior of primary health providers and their performance on continuity and coordination of diabetes patient management care.

To fill this gap, this study aimed to explore the impact of the performance assessment practices, including adding requirement of CFDS on performance assessment and linking the income with the assessment results on CFDS, on performance of diabetes patient management care in China.

## Methods

### Study Design and Setting

Because in China the health system development status is closely related to local economic development, which is then closely related to the geographic position. During the process of choosing sample cities we assumed the diversity in regions could represent the different primary health system situtions in China. In addition, we also consider including those cities where the CFDS have been piloted, so finally, six cities were chosen as sample cities, including Tongren, Xining, Shenzhen, Xiamen, Fuyang and Suzhou. For the sampling of PHC facilities, we used multi-stage cluster random sampling method: two districts/counties were be randomly selected in each of the six cities; in each district/county, 12 PHC facilities were randomly selected, including 6 community healthcare centers in urban areas and 6 township health centers in rural areas; if there was no any rural counties under the jurisdiction of one city, 12 community health centers were randomly selected. All the on duty health workers on the investigation day were included in the survey, including doctors, nurses, and public health workers. All the diabetes patients visiting PHC facilities for outpatient care on the investigation day were also interviewed. Finally, 72 PHC facilities (47 community health centers, 25 township health centers), 827 PHC health workers and 420 diabetes patients were included in the analysis.

### Measurement

In economic theory, the work behaviors of health workers involve of costs to themselves (exhaustion from efforts); at the same time their work behaviors also directly or indirectly contribute to the delivery of health services to patients, for which the purchasers or employers transfering money to compensate health workers, and then the monetary rewards bring benefits to them. In traditional economic terms, as human beings, health workers are viewed as maximizing their utility function (23). As monetary rewards are one of the most important factors increasing utility, health professionals' behavior change is to try to reduce the ratio of the costs and benefits (24). Based on this theory, we assume that linking the income of health workers with their performance assessment results will motivate the health workers to input more for better performance and then maximizing their income level.

#### Dependent Variables

This study analyzed two levels of dependent variables on the process and outcome quality indicators of diabetes patient management care, including the diabetes patient management care delivery process of individual PHC health workers, diabetes patients' outcomes in the utilization of services and self-reported blood glucose control at the level of patients.

The process quality of diabetes patient management care focused on the continuity and coordination of care. The continuity of care was measured by “whether or not the health workers could follow the relevant information on the diabetes patients,” including “medical history” (1 = yes, 0 = no), “status of blood glucose control” (1 = yes, 0 = no), “lifestyle pattern” (1 = yes, 0 = no), “treatment plan at PHC facility” (1 = yes, 0 = no), and “treatment plan at other institutions” (1 = yes, 0 = no). Six items were summed to create the number of categories of patients' information which were followed by the doctors or other health workers, and this summed variable ranged from 0 to 5. The coordination of care was measured by the extent of information sharing on diabetes patient management care between departments within the facility (1 = good, and 0 = bad), and the sharing of patients' information on diabetes management between the PHC facility and other institutions (1 = good, and 0 = bad).

The outcome performance of diabetes patient management care was also measured by the diabetes patients' utilization of services and self-reported health outcome. The utilization of services was assessed by the categories of services received by patients, including medicine consultation (1 = yes, 0 = no), physical examination (1 = yes, 0 = no), biochemical examination (1 = yes, 0 = no), blood glucose testing (1 = yes, 0 = no), formulating a emergency treatment plan (1 = yes, 0 = no), making a lifestyle modification plan (1 = yes, 0 = no), health education (1 = yes, 0 = no), management of medical records (1 = yes, 0 = no), and making an appointment for follow-up (1 = yes, 0 = no). These nine variables were summed to create the number of service category received by patients, ranging from 0 to 9. The health outcome performance was measured by blood glucose control status reported by patients with the code of 1 indicating that patients achieved glycemic control, and the code of 0 indicating that patients had not achieved glycemic control.

#### Independent Variables

The key independent variable analyzed in this study is related to the practice of performance assessment practices for health workers in PHC facilities, which was measured by whether the performance of CFDS is added into the overall performance assessment system (yes or no), and whether the performance of CFDS was linked with the increase of personal income of PHC providers (yes or no).

The influence of PBS design on delivery process performance was analyzed at individual level, and the analysis on patients' utilization and health outcomes was measured at facility level. In order to measure the level of incentives for CFDS in facility level, i.e., the inclusion of CFDS performance in performance assessment system and the linkage between health workers income with their CFDS performance, we assumed that more health workers perceiving the inclusion of the CFDS performance and more perceiving linkage between their income and performance on CFDS, as the stronger level of incentives. So we imputed the percentage of health workers who reported performance on CFDS being included in the overall performance assessment in a facility, and this imputed variable was divided into trisections (low-, middle-, or high-level). We also imputed the percentage of health workers who reported their performance on CFDS increasing their income, and this imputed variable was also divided into three equal parts (low-, middle-, or high-level).

#### Control Variables

In the individual level analysis, control variables were sociodemographic characteristics of health workers, including sex (male or female), age (<30, 30–40, 40–55, or >55), educational background (high school and below, junior college, bachelor, or master and above), and employment status (permanent or temporary). In the facility level analysis, sociodemographic characteristics of diabetes patients were also added as other control variables, including sex (male or female), age (<45, 45–60, 60–75, or >75), educational background (illiteracy, primary school, junior high school, high school and technical secondary school, or junior college and above), health insurance (yes or no) and having other chronic diseases (yes or no).

### Statistical Analysis

We conducted descriptive analyses to examine the characteristics of PHC health workers and diabetes patients. Chi-square tests were used to test the differences in socio-demographic characteristics of health workers and patients with diabetes. Multivariate logistic regression models at the unit of individual health workers were used to investigate the association between the incentives on family doctor contract services and the continuity and coordination of diabetes patient management care. Multilevel multivariate regression models at the unit of health facility were used to investigate the association between PBS incentives for family doctor contract services and patients' utilization of management care and blood glucose control. To account for unmeasured variations within each facility, we applied multilevel random intercept analyses to process the two-level structure of patient-level and institutional-level data. All the models were adjusted for control variables reported by coefficients (Coef.) or odds ratios (ORs) with corresponding 95% confidence intervals (CI). All statistical analyses were conducted using Stata V.15.0.

## Results

### Descriptive Results

Table 1 presents the characteristics of PHC health workers. Among the 827 investigated health workers, the majority were female (77.0%), had education level of bachelor (64.6%), and had permanent employment status (63.2%). About 87.8% of investigated health workers reported that their performance on CFDS has been added into the overall performance assessment, and 57.9% reported that their income has ever been increased because their performance on CFDS. For PHC health workers whose performance on CFDS was added in assessment, they performed better on three performance indicators of diabetes patient management care than the health workers whose provision of contracted services were not assessed: the score of continuity of care (4.323 vs. 3.969, *p* = 0.005), the percentage of good sharing information within facility (92.5 vs. 86.4%, *p* = 0.050), the percentage of good sharing information between institutions (78.7 vs. 68.9%, *p* = 0.036). PHC health workers whose performance on contracted services had linked to the increase of their income had higher score of continuity of care (4.362, 4.136 vs. 4.168, *p* = 0.064), and had higher percentage of good sharing information within institution (95.1, 85.0 vs. 87.4%, *p* = 0.001), than whose performance on contracted services did not change their income.

**Table 1:** Characteristics of primary healthcare providers.

	**Total, *n* (%)**	**Mastery of patient's information, mean (SD)**	***P***	**Information sharing within institution, *n* (%)**	***P***	**Information sharing with other institutions, *n* (%)**	***P***
**Sex**							
Male	190 (23.0%)	4.335 (1.047)	0.504	155 (87.1%)	0.010	127 (73.4%)	0.145
Female	637 (77.0%)	4.269 (1.200)		546 (93.2%)		454 (78.7%)	
**Age**							
<30	244 (29.5%)	4.170 (1.214)	0.100	197 (90.0%)	0.128	175 (81.0%)	0.472
30–40	401 (48.5%)	4.379 (1.130)		345 (92.3%)		278 (76.0%)	
40–50	147 (17.8%)	4.172 (1.210)		135 (95.1%)		105 (75.5%)	
>50	35 (4.2%)	4.364 (1.055)		26 (83.9%)		25 (80.7%)	
**Educational background**							
High school and below	32 (3.9%)	4.063 (1.343)	<0.001	28 (93.3%)	0.503	28 (90.3%)	0.029
Junior college	213 (25.8%)	4.043 (1.301)		182 (91.5%)		160 (82.1%)	
Bachelor	534 (64.6%)	4.343 (1.118)		450 (91.3%)		365 (75.9%)	
Master and above	48 (5.8%)	4.783 (0.664)		43 (97.7%)		30 (66.7%)	
**Employment status**							
Temporary	274 (33.1%)	4.170 (1.229)	0.157	239 (92.3%)	0.483	206 (81.8%)	0.138
Permanent	523 (63.2%)	4.331 (1.151)		440 (91.9%)		356 (75.3%)	
NA	30 (3.6%)	4.413 (0.825)		24 (85.7%)		21 (77.8%)	
**Whether the performance of contracted service is included in the overall performance assessment**							
No	101 (12.2%)	3.969 (1.439)	0.005	76 (86.4%)	0.050	62 (68.9%)	0.036
Yes	726 (87.8%)	4.323 (1.121)		627 (92.5%)		521 (78.7%)	
**How the performance of contracted service influences the personal income**							
None	325 (39.3%)	4.168 (1.205)	0.064	264 (87.4%)	0.001	223 (74.1%)	0.163
Decrease in income	23 (2.8%)	4.136 (1.246)		17 (85.0%)		16 (84.2%)	
Increase in income	479 (57.9%)	4.362 (1.136)		422 (95.1%)		344 (79.6%)	

Table 2 presents the characteristics of diabetes patients. Among the 420 patients, 54.2% were female, 53.3% were between the ages of 60 and 75, 97.9% had health insurance, and 76.6% had other chronic diseases. Table 3 presents the patients' utilization of diabetes management care, the self-reported control of blood glucose, and the facility-level incentive design of PBS for motivating the contracted service. The percentages of the low, middle, and high level of health workers who reported CFDS performance being included in the overall performance assessment were 32.4, 33.3, and 34.3%, respectively. The same level of percentage of health workers who reported their income being increased because of CFDS performance were 11.4, 55.9, and 32.6%, respectively. For PHC facilities with high percentage of health workers who reported performance on contracted service being assessed, the rate of patients with blood glucose under control was higher (82.6%) than the rate of patients covered by facilities with middle (77.4%) and low (67.2%) level percentages of health workers who has been assessed regarding the contracted services (*p* = 0.011). For PHC facilities with high percentage of health workers who reported performance on CFDS being linked with increased income, the score of patients' utilizations of diabetes treatment care were higher (Mean 6.72, SD 1.96) than those with middle (Mean 5.94, SD 1.96) and low (Mean 5.53, SD 2.16) level percentages of health workers whose income level was linked with the performance on CFDS (*p* < 0.001).

**Table 2:** Characteristics of diabetic patients.

	**Total, *n* (%)**	**Diabetic treatment, mean (SD)**	***P***	**Blood sugar control, *n* (%)**	***P***
**Sex**					
Male	192 (45.8%)	6.200 (2.194)	0.718	145 (78.4%)	0.266
Female	228 (54.2%)	6.121 (2.191)		159 (73.6%)	
**Age**					
<45	21 (5.0%)	6.238 (2.234)	0.371	15 (75.0%)	0.998
45–60	113 (26.9%)	5.963 (2.236)		83 (76.2%)	
60–75	224 (53.3%)	6.318 (2.162)		162 (76.1%)	
>75	62 (14.8%)	5.864 (2.177)		45 (75.0%)	
**Educational background**					
Illiteracy	95 (22.7%)	6.032 (2.113)	0.945	66 (73.3%)	0.731
Primary school	104 (24.8%)	6.097 (2.176)		80 (80.0%)	
Junior high school	115 (27.5%)	6.252 (2.226)		81 (73.0%)	
High school and technical secondary school	70 (16.7%)	6.212 (2.324)		52 (77.6%)	
Junior college and above	35 (8.4%)	6.286 (2.136)		26 (78.8%)	
**Health insurance**					
No	9 (2.1%)	6.667 (1.732)	0.478	7 (77.8%)	0.892
Yes	411 (97.9%)	6.142 (2.198)		298 (75.8%)	
**Having other chronic diseases**					
No	98 (23.4%)	6.505 (2.123)	0.075	78 (83.0%)	0.062
Yes	320 (76.6%)	6.048 (2.201)		225 (73.5%)	

**Table 3:** Facility-level incentive of performance-based salary system and patient-level diabetes services received and blood glucose control.

	**Total, *n* (%)**	**Diabetic treatment, mean (SD)**	***P***	**Blood sugar control, *n* (%)**	***P***
**Percentage of health workers who reported CFDS performance being included in the overall performance assessment**					
Low	136 (32.4%)	6.18 (2.21)	0.387	88 (67.2%)	0.011
Middle	140 (33.3%)	5.96 (2.19)		103 (77.4%)	
High	144 (34.3%)	6.31 (2.17)		114 (82.6%)	
**Percentage of health workers who reported their income being increased because of CFDS performance**					
Low	48 (11.4%)	5.53 (2.16)	<0.001	34 (72.3%)	0.074
Middle	235 (55.9%)	5.94 (2.26)		164 (72.6%)	
High	137 (32.6%)	6.72 (1.96)		107 (82.9%)	

### Association Between Performance Assessment Methods and Performance on Diabetes Patient Management Care

Associations between how performance assessment was designed and the continuity and coordination of care at the individual level of health worker are shown in Table 4. PHC health workers with the performance on CFDS being assessed had 0.279 (Coef. 0.279, 95% CI 0.031–0.526, *p* = 0.028) higher score on the continuity of care, and had 92.6% (OR 1.926, 95% CI 1.160–3.197; *p* = 0.011) higher likelihood of sharing information on patients between institutions. PHC health workers whose income has been increased because of performance on contracted services were 168.1% (OR 2.681, 95% CI 1.502–4.788; *p* = 0.001) and 78.0% (OR 1.780, 95% CI 1.220–2.597; *p* = 0.003) more likely to share information on patients within institution and between institutions, respectively.

**Table 4:** Associations between performance-based salary system incentive and care continuity and coordination among primary healthcare workers.

**Mastery of patients' information**			**Information sharing within institution**			**Information sharing with other institutions**		
**Coef**.	**95% CI**	***P***	**OR**	**95% CI**	***P***	**OR**	**95% CI**	***P***
0.279	(0.031, 0.526)	0.028	1.823	(0.909, 3.655)	0.091	1.926	(1.160, 3.197)	0.011
−0.035	(−0.539, 0.468)	0.890	0.740	(0.197, 2.781)	0.656	1.820	(0.490, 6.763)	0.371
0.093	(−0.080, 0.265)	0.293	2.681	(1.502, 4.788)	0.001	1.780	(1.220, 2.597)	0.003

Associations between how performance assessment was designed and performance on diabetes patient management care at facility level are shown in Table 5. Additional one-point percentage of health workers who reparted performance on CFDS being assessed was associated with 7.192 (OR 8.192, 95% CI 1.903–35.266; *p* = 0.005) times of probability of more covered patients with blood glucose under control. Additional one-point percentage of health workers who reported their income being increased because of performance on contracted services was associated with 52.2% (OR 1.522, 95% CI 1.055–2.196; *p* = 0.025) probability of more covered patients having blood glucose under control. Additional one-point percentage of PHC health workers who reported their income being increased was associated with 0.559 (Coef. 0.559, 95% CI 0.139–0.979; *p* = 0.009) more score on the covered patients' utilization of management care.

**Table 5:** Associations between performance-based salary system incentive and performance on diabetes patient management care at facility level.

**Diabetic treatment**			**Blood sugar control**		
**Coef**.	**95% CI**	***P***	**OR**	**95% CI**	***P***
0.272	(−1.356, 1.900)	0.743	8.192	(1.903, 35.266)	0.005
0.559	(0.139, 0.979)	0.009	1.522	(1.055, 2.196)	0.025

## Discussion

To the best of our knowledge, this study was the first to examine the relationship between the CFDS-related performance assessment and incentives for health workers in PHC facilities and their performance on diabetes patient management care. The key findings are the inclusion of performance of contracted service in the overall performance assessment was associated with the increasing the continuity and coordination of diabetes patient management care. Health workers with experience of increasing income because of the performance on contracted service was associated with better provision of care and more patients with blood glucose under control.

In China, PHC facilities are not the point of first contact, and the residents, including the diabetes patients can choose bypass PHC facilities to seek healthcare at high-level hospitals, which leads to escalating medical costs and low efficiency of whole health system (25). The implementation of CFDS is a critical way with the intention to change the traditional delivery pattern of China's PHC services (26–28). CFDS policy tries to build the multidisciplinary team, construct the stable relationship with patients, and improve the quality of PHC. As PHC facilities have autonomy in designing the detailed assessment criteria and payment methods for their staff, some facilities have started to use incentives to encourage the development of family doctor teams and quality of contracted services. Furthermore, in the pilots of contracted services, the first group of residents to be covered by contracted service all targeted the chronic disease patients, i.e., diabetes patients and high blood-pressure patients. Thus, this study used the opportunity produced by different implementation status in different facilities, chose the outcomes in diabetes patient management care, and analyzed whether the performance assessment and incentives for CFDS were related on the performance of diabetes patient management care.

Lack of financial incentive for PHC providers is one of the causes for the poor quality of PHC (29). First, the income level of health workers in PHC facilities was only about 30–50% of their expected pay level. Second, regarding the payment method, i.e., the percentage of performance-based bonus on the total income is low, which has limited incentive power to guide the behavior of health worker (30). The CFDS policy tries to target these problems. The national policy guidance on the CFDS requires that PHC facilities include the contents of family doctor services into the performance assessment criteria of health workers, and increase the performance-related income to those with more contracted patients and better performance on contracted services. However, the implementation extent of different areas, different facilities, and different family doctor teams is different. In addition, the facility managers are in charge of design for the performance criteria and the performance target for their personnel. These varied situation in implementation process and among different facilities provided good opportunity for us to study the relationship between the added incentives for contracted services and performance of family doctor team members.

This study found that the performance on continuity and coordination of diabetes patient management care were positively related to inclusion of CFDS requirement into performance assessment. The reasons can be found and understood from the aims and design of CFDS policy, and the measurement methods for the continuity and coordination of services in this study were designed based on the service contents and procedures defined in these policy documents.

Firstly, the contents of services contracted by family doctor team are designed by policy makers with the intention to solve the lack of continuity in PHC. Health system in China is featured by the fragmentation among different tiers of delivery institutions, PHC facilities are not the gatekeeper and patients have a strong preference to bypass the PHC facilities in favor of hospitals. As consequence, few patients contacted the same primary health care worker who was familiar with them and had long-term follow-up from this health worker, which is a barrier to improve the quality of chronic disease care. In national guideline of CFDS, some services are listed to strengthen the close relationship between family doctor team and patients, including more frequent follow-up, individualized education, long-term prescription, home visits, prioritized referral etc., all of which can help build the long-term and continuous caring provided by the same family doctor team and could result in family doctors team being more familiar with patients and their health care seeking history. So in this study continuity of care was measured by the awareness of health workers on the treatment and management experience of patients.

Secondly, the contents of services and the organization of family doctor team required by CFDS policy directly targeting the lack of coordination in health care for diabetes patients. The services provided by PHC facilities inside were fragmented in traditionally. The medical services were provided by physicians and nurses; while preventive and management care were provided by public health workers (31). For instance, in diabetes management visits under the National Basic Public Health Service Program, patients can only have blood glucose measurement and lifestyle consultations by public health workers, but when patients need prescriptions of hypoglycemic drugs, they had to contact doctors separately (12). Furthermore, the electronic medical record system at PHC facilities was commonly fragmented, with public health workers documenting management records in public health services system and doctors prescribing medicines in hospital information system (12). National government policy guidance documents listed some minimum requirements on the package of contracted services, including continuous services from common disease treatment, basic public health services, health management, health education and consultation, referral to hospitals, etc. Furthermore, care for chronic disease patients need coordination of treatment and management services provided by different levels of health providers. The CFDS policy also required that as the major health manager for the contracted chronic disease patients, the family doctor team and its members have the responsibility in coordinating these services for their patients. All these designs were intended to solve the above fragmentation problems, especially the national policy guideline recommends use of information integration and telemedicine technologies to facilitate the coordination (8, 32). That is why this study use the level of information sharing inside the PHC facility and the level of information sharing between PHC facilities and hospitals as the measurements of coordination of care.

The finding on the positive relationship between incentive and better performance is consistent with the basic hypothesis that health workers seek to maximize the utility in the selection of work behaviors and the utility of health workers depends on their income and the health status of the contracted patients (31). The incentive links quality of care by PHC health workers with their income, which guides PHC health workers seeking to increase their income through providing care of high quality to maximize their utility. Prior empirical studies also have the similar findings that the introduction of pay-for-performance incentive and linking remuneration for general practices to recorded quality of care for diabetes, can increase the provision performance of healthcare services (15–17).

In addition to the positive influence on provision behavior of PHC health workers, this study also find that the incentive is related to positive results on performance measured from the patients' service utilization and health outcomes. This positive relationship may be explained through the better continuity of services contributing to more utilization of services and then better health outcomes. There have been some evidences on the association between higher continuity of care and better health outcomes among diabetes patients. Several studies found that higher continuity of care was associated with more services used by diabetes patients, including more HbA1 testing and eye or foot examinations (33, 34). Continuity of care can also facilitate higher patient self-care behaviors, compliance and adherence to physicians' recommendations, which could be the reason for better health outcomes (35). However, the results of some other studies had different findings. For instance, prior studies did not generate evidence supporting a beneficial effect of the pay-for-performance incentive on treatment (e.g., rate of HbA1c test, rate of lipid test, rate of dilated eye exam) and control of blood glucose (11, 15, 36). A synthesis result based on high-quality studies on effects of pay-for-performance incentives also found that the pay-for-performance incentive can only have impacts on service procedure outcomes, but not on the patient outcome (37). One possible reason is that our study used the self-reported service utilization and blood glucose control to measure the performance, which probably over-estimated the level of performance. Another possible reason is that the duration of incentives might be different in different studies, and the power of incentives is usually weakened over time, and in China the introduction of family doctor services and relevant incentives for PHC health workers just started (28).

### Limitations

This study has several limitations. First, the observational nature of our study limited our ability to draw any causal inference from our findings. The results should not be interpreted as the effect of adding incentive on diabetes patient management care processes and outcomes. Rather, the association found in this study underscored the need for research to develop financial incentive policy to improve diabetes management. Future studies should focus on more rigorous research, including randomized, controlled trials and observational studies with concurrent control groups, to assess the effectiveness of the incentive among the CFDS. And the longitudinal study and ongoing monitoring of the incentive program is critical to determine the effectiveness of incentives and the possible unintended effects on diabetes patients and health care providers. Secondly, the measurements of continuity and coordination of care were based on self-developed questions and only used continuity and sharing status in patients' information as the proxy indicators. Thirdly, the measurements of blood glucose control were performed by self-administrated method, which may lead to the self-administrated bias. Finally, the performance assessment in our study was for all CFDS rather than specifically diabetes management care. However, the association between the incentive and performance on diabetes patient management care provide in-depth explanation and reliable support for policy importance to diabetes population in China.

## Conclusions

The incentive linking income with performance on the CFDS in China PHC setting is probably positively associated with process and outcome performance on diabetes patient management care. Design and implementation of the incentive should be accompanied with the policy of CFDS, in order to increase the proportion of performance-related income of PHC providers, thereby improving the quality of diabetes patient management care and health status of diabetes patients.

## Data Availability Statement

The raw data supporting the conclusions of this article will be made available by the authors, without undue reservation.

## Ethics Statement

The study has been approved by the Ethics Review Board of the School of Public Health, Peking University. Informed consent was obtained from all participants prior to questionnaire administration. All methods were carried out in accordance with relevant guidelines and regulations under ethics approval and consent to participate. The patients/participants provided their written informed consent to participate in this study.

## Author Contributions

BY developed the study design. WT and YJ conducted data analysis and interpretation. YJ drafted the manuscript. BY reviewed the manuscript and provided revisions. All authors read and approved the final manuscript.

## Funding

This work was supported by the Natural Science Foundation of Beijing (grant number 9202007).

## Conflict of Interest

The authors declare that the research was conducted in the absence of any commercial or financial relationships that could be construed as a potential conflict of interest.

## Publisher's Note

All claims expressed in this article are solely those of the authors and do not necessarily represent those of their affiliated organizations, or those of the publisher, the editors and the reviewers. Any product that may be evaluated in this article, or claim that may be made by its manufacturer, is not guaranteed or endorsed by the publisher.

## Abbreviations

CFDS: contracted family doctor service
PBS: Performance-Based Salary
PHC: Primary Healthcare
CI: Confidence Interval
OR: Odds Ratios.
