# Supplementary material for: Incentives Promoting Contracted Family Doctor Service Policy to Improve Continuity and Coordination in Diabetes Patient Management Care in China
Source: Front Public Health. 2022 Jul 15;10:843217. doi: 10.3389/fpubh.2022.843217 (PMC9334846; doi:10.3389/fpubh.2022.843217)
Supplement: Supplementary file 1 [file Table_1.DOCX]

**Appendix Table 1.** Inclusion of CFDS performance and provision of diabetes care at PHC- provider level

|  | Mastery of patients' information | | |  | Information sharing within institution | | | |  | Information sharing with other institutions | | | | |
| --- | --- | --- | --- | --- | --- | --- | --- | --- | --- | --- | --- | --- | --- | --- |
|  | Coefficient | 95% CI | P |  | Coefficient | OR | 95% CI | P |  | Coefficient | OR | 95% CI | P |  |
| Whether the performance of contracted service is included in the overall performance assessment (reference, no) |  |  |  |  |  |  |  |  |  |  |  |  |  |  |
| Yes | 0.279 | [0.031, 0.526] | 0.028 |  | 0.600 | 1.823 | [0.909, 3.655] | 0.091 |  | 0.655 | 1.926 | [1.160, 3.197] | 0.011 |  |
| Sex (reference, male) |  |  |  |  |  |  |  |  |  |  |  |  |  |  |
| Female | -0.070 | [-0.266, 0.127] | 0.485 |  | 0.799 | 2.224 | [1.254, 3.946] | 0.006 |  | 0.327 | 1.386 | [0.916, 2.098] | 0.122 |  |
| Age (reference, <30) |  |  |  |  |  |  |  |  |  |  |  |  |  |  |
| 30-40 | 0.028 | [-0.194, 0.249] | 0.806 |  | 0.328 | 1.388 | [0.686, 2.809] | 0.362 |  | 0.143 | 1.153 | [0.689, 1.932] | 0.587 |  |
| 40-55 | -0.114 | [-0.424, 0.196] | 0.471 |  | 1.036 | 2.817 | [0.919, 8.631] | 0.070 |  | 0.412 | 1.510 | [0.744, 3.062] | 0.253 |  |
| >55 | 0.135 | [-0.341, 0.610] | 0.578 |  | -0.397 | 0.672 | [0.185, 2.441] | 0.546 |  | 0.480 | 1.616 | [0.527, 4.957] | 0.401 |  |
| Educational background (reference, High school or below) |  |  |  |  |  |  |  |  |  |  |  |  |  |  |
| Junior college | -0.074 | [-0.532, 0.385] | 0.753 |  | -0.314 | 0.731 | [0.144, 3.701] | 0.705 |  | -0.545 | 0.580 | [0.159, 2.118] | 0.410 |  |
| Bachelor | 0.109 | [-0.356, 0.574] | 0.645 |  | -0.444 | 0.641 | [0.126, 3.274] | 0.593 |  | -0.667 | 0.513 | [0.141, 1.863] | 0.311 |  |
| Master and above | 0.525 | [-0.048, 1.098] | 0.073 |  | 1.005 | 2.731 | [0.209, 35.711] | 0.444 |  | -1.117 | 0.327 | [0.078, 1.375] | 0.127 |  |
| Employment status (reference, temporary) |  |  |  |  |  |  |  |  |  |  |  |  |  |  |
| Permanent | -0.013 | [-0.201, 0.175] | 0.892 |  | 0.098 | 1.103 | [0.595, 2.046] | 0.755 |  | -0.007 | 0.993 | [0.653, 1.509] | 0.973 |  |
| NA | 0.164 | [-0.280. 0.609] | 0.469 |  | -0.427 | 0.652 | [0.196, 2.169] | 0.486 |  | 0.007 | 1.010 | [0.371, 2.729] | 0.989 |  |

**Appendix Table 2.** Increase in income by CFDS performance assessment and provision of diabetes care at PHC-provider level

|  | Mastery of patients' information | | |  | Information sharing within institution | | | |  | Information sharing with other institutions | | | | |
| --- | --- | --- | --- | --- | --- | --- | --- | --- | --- | --- | --- | --- | --- | --- |
|  | Coefficient | 95% CI | P |  | Coefficient | OR | 95% CI | P |  | Coefficient | OR | 95% CI | P |  |
| How the performance of contracted service influences the personal income (reference, no change in income) |  |  |  |  |  |  |  |  |  |  |  |  |  |  |
| Decrease in income | -0.035 | [-0.539, 0.468] | 0.890 |  | -0.301 | 0.740 | [0.197, 2.781] | 0.656 |  | 0.599 | 1.820 | [0.490, 6.763] | 0.371 |  |
| Increase in income | 0.093 | [-0.080, 0.265] | 0.293 |  | 0.986 | 2.681 | [1.502, 4.788] | 0.001 |  | 0.576 | 1.780 | [1.220, 2.597] | 0.003 |  |
| Sex (reference, male) |  |  |  |  |  |  |  |  |  |  |  |  |  |  |
| Female | -0.068 | [-0.265, 0.129] | 0.497 |  | 0.765 | 2.150 | [1.205, 3.835] | 0.010 |  | 0.315 | 1.370 | [0.905, 2.075] | 0.137 |  |
| Age (reference, <30) |  |  |  |  |  |  |  |  |  |  |  |  |  |  |
| 30-40 | 0.039 | [-0.183, 0.262] | 0.728 |  | 0.366 | 1.441 | [0.701, 2.963] | 0.320 |  | 0.148 | 1.159 | [0.690, 1.948] | 0.575 |  |
| 40-55 | -0.094 | [-0.405, 0.217] | 0.553 |  | 1.134 | 3.107 | [0.991, 9.738] | 0.052 |  | 0.447 | 1.563 | [0.767, 3.185] | 0.218 |  |
| >55 | 0.141 | [-0.336, 0.620] | 0.561 |  | -0.389 | 0.678 | [0.182, 2.528] | 0.562 |  | 0.445 | 1.559 | [0.510, 4.776] | 0.436 |  |
| Educational background (reference, High school or below) |  |  |  |  |  |  |  |  |  |  |  |  |  |  |
| Junior college | -0.085 | [-0.544, 0.375] | 0.718 |  | -0.392 | 0.675 | [0.129, 3.523] | 0.641 |  | -0.611 | 0.543 | [0.148, 1.991] | 0.357 |  |
| Bachelor | 0.102 | [-0.364, 0.569] | 0.667 |  | -0.607 | 0.545 | [0.103, 2.885] | 0.475 |  | -0.742 | 0.476 | [0.131, 1.736] | 0.261 |  |
| Master and above | 0.524 | [-0.053, 1.100] | 0.075 |  | 0.649 | 1.914 | [0.140, 26.133] | 0.627 |  | -1.271 | 0.281 | [0.066, 1.194] | 0.085 |  |
| Employment status (reference, temporary) |  |  |  |  |  |  |  |  |  |  |  |  |  |  |
| Permanent | -0.015 | [-0.204, 0.173] | 0.872 |  | 0.145 | 1.156 | [0.622, 2.148] | 0.647 |  | -0.032 | 0.969 | [0.637, 1.473] | 0.882 |  |
| NA | 0.165 | [-0.282, 0.611] | 0.469 |  | -0.424 | 0.654 | [0.191, 2.243] | 0.500 |  | -0.004 | 0.996 | [0.368, 2.699] | 0.994 |  |

**Appendix Table 3.** Institutional-level percentage of whose CFDS performance included in assessment and diabetic care of patients

|  | Diabetic treatment | | |  | Blood sugar control | | | |
| --- | --- | --- | --- | --- | --- | --- | --- | --- |
|  | Coefficient | 95% CI | P |  | Coefficient | OR | 95% CI | P |
| **Institutional level** |  |  |  |  |  |  |  |  |
| Percentage of whose performance of contracted service included in the overall performance assessment | 0.272 | [ -1.356, 1.900] | 0.743 |  | 2.103 | 8.192 | [1.903, 35.266] | 0.005 |
| **Patient level** |  |  |  |  |  |  |  |  |
| Sex (reference, male) |  |  |  |  |  |  |  |  |
| Female | -0.105 | [-0.590, 0.381] | 0.672 |  | -0.405 | 0.667 | [0.405, 1.099] | 0.112 |
| Age (reference, <45) |  |  |  |  |  |  |  |  |
| 45-60 | -0.081 | [-1.173, 1.010] | 0.884 |  | 0.412 | 1.511 | [0.475, 4.805] | 0.485 |
| 60-75 | 0.151 | [-0.907, 1.209] | 0.780 |  | 0.344 | 1.410 | [0.480, 4.1456] | 0.532 |
| >75 | -0.130 | [-1.267, 1.008] | 0.823 |  | 0.426 | 1.531 | [0.480, 4.886] | 0.472 |
| Educational background (reference, illiteracy) |  |  |  |  |  |  |  |  |
| Primary school | -0.008 | [-0.615, 0.600] | 0.980 |  | 0.176 | 1.193 | [0.535, 2.661] | 0.667 |
| Junior high school | 0.040 | [-0.577, 0.657] | 0.900 |  | -0.428 | 0.652 | [0.333, 1.274] | 0.211 |
| High school and technical secondary school | 0.073 | [-0.777, 0.922] | 0.867 |  | -0.223 | 0.800 | [0.321, 1.995] | 0.632 |
| Junior college and above | -0.135 | [-1.009, 0.739] | 0.762 |  | -0.144 | 0.866 | [0.306, 2.449] | 0.786 |
| Health insurance (reference, no) |  |  |  |  |  |  |  |  |
| Yes | -0.603 | [-1.887, 0.681] | 0.357 |  | 0.536 | 1.709 | [0.249, 11.716] | 0.586 |
| Having other chronic diseases (reference, no) |  |  |  |  |  |  |  |  |
| Yes | -0.237 | [-0.705, 0.232] | 0.322 |  | -0.599 | 0.550 | [0.295, 1.024] | 0.059 |

**Appendix Table 4.** Institutional-level percentage of whose income increased by CFDS performance assessment and diabetic care of patients

|  | Diabetes treatment | | |  | Blood sugar control | | | |
| --- | --- | --- | --- | --- | --- | --- | --- | --- |
|  | Coefficient | 95% CI | P |  | Coefficient | OR | 95% CI | P |
| **Institutional level** |  |  |  |  |  |  |  |  |
| Percentage of whose performance of contracted service increases the personal income | 0.559 | [0.139, 0.979] | 0.009 |  | 0.420 | 1.522 | [1.055, 2.196] | 0.025 |
| **Patient level** |  |  |  |  |  |  |  |  |
| Sex (reference, male) |  |  |  |  |  |  |  |  |
| Female | -0.119 | [-0.601, 0.362] | 0.628 |  | -0.364 | 0.695 | [0.425, 1.136] | 0.147 |
| Age (reference, <45) |  |  |  |  |  |  |  |  |
| 45-60 | -0.090 | [-1.191, 1.011] | 0.872 |  | 0.319 | 1.376 | [0.428, 4.421] | 0.592 |
| 60-75 | 0.125 | [0-.926, 1.176] | 0.815 |  | 0.242 | 1.273 | [0.437, 3.709] | 0.658 |
| >75 | -0.114 | [-1.236, 1.008] | 0.842 |  | 0.333 | 1.395 | [0.441, 4.413] | 0.571 |
| Educational background (reference, illiteracy) |  |  |  |  |  |  |  |  |
| Primary school | -0.108 | [-0.728, 0.512] | 0.734 |  | 0.200 | 1.221 | [0.559, 2.668] | 0.616 |
| Junior high school | -0.018 | [-0.630, 0.594] | 0.954 |  | -0.396 | 0.673 | [0.350, 1.291] | 0.233 |
| High school and technical secondary school | -0.061 | [-0.911, 0.790] | 0.889 |  | -0.213 | 0.808 | [0.324, 2.014] | 0.647 |
| Junior college and above | -0.223 | [-1.102, 0.657] | 0.620 |  | -0.069 | 0.933 | [0.338, 2.579] | 0.893 |
| Health insurance (reference, no) |  |  |  |  |  |  |  |  |
| Yes | -0.411 | [-1.632, 0.811] | 0.510 |  | 0.602 | 1.827 | [0.236, 14.113] | 0.564 |
| Having other chronic diseases (reference, no) |  |  |  |  |  |  |  |  |
| Yes | -0.191 | [-0.662, 0.280] | 0.427 |  | -0.611 | 0.543 | [0.290, 1.015] | 0.056 |
